# Supplementation of Yupingfeng polysaccharides in low fishmeal diets enhances intestinal health through influencing the intestinal barrier, immunity, and microflora in *Macrobrachium rosenbergii*

**DOI:** 10.3389/fimmu.2024.1480897

**Published:** 2024-11-26

**Authors:** Mingyang Liu, Cunxin Sun, Qunlan Zhou, Pao Xu, Aimin Wang, Xiaochuan Zheng, Bo Liu

**Affiliations:** ^1^ Key Laboratory of Freshwater Fisheries and Germplasm Resources Utilization, Ministry of Agriculture and Rural Affairs, Freshwater Fisheries Research Center, Chinese Academy of Fishery Sciences, Wuxi, China; ^2^ Wuxi Fisheries College, Nanjing Agricultural University, Wuxi, China; ^3^ College of Marine and Biology Engineering, Yancheng Institute of Technology, Yancheng, China

**Keywords:** Yupingfeng polysaccharides, alternative fish meal diet, peritrophic matrix, intestinal immunity, intestinal microbes, *Macrobrachium rosenbergii*

## Abstract

**Introduction:**

This study aimed to investigate the effects of a low-fishmeal diet (LF, substituting soybean meal for 40% fish meal) and the supplementation of 500 mg/kg and 1000 mg/kg Yu Ping Feng (YPF) polysaccharides on the growth performance, antioxidant enzyme activities, intestinal ultrastructure, non-specific immunity, and microbiota of *Macrobrachium rosenbergii*.

**Methods:**

The study involved the administration of different diets to M. rosenbergii, including a control diet, a low-fishmeal diet (LF), and LF diets supplemented with 500 mg/kg and 1000 mg/kg YPF polysaccharides. Growth performance, antioxidant enzyme activities, intestinal ultrastructure, non-specific immunity, and microbiota were assessed.

**Results:**

The LF diet significantly reduced growth performance parameters compared to the control group. However, YPF supplementation notably improved these parameters, with the greatest improvement observed at a 1000 mg/kg dosage. Antioxidant enzyme activities (SOD, GSH-PX) were diminished in the LF group, accompanied by elevated MDA levels, whereas YPF supplementation restored these activities and reduced MDA levels. Ultrastructural analysis revealed that the LF diet caused intestinal villi detachment and peritrophic matrix (PM) shedding, which were alleviated by YPF. Gene expression related to PM formation (GS, CHS, EcPT) was downregulated in the LF group but significantly upregulated in the 1000P group. Non-specific immune gene expressions (IMD, Relish, IκBα) and enzyme activities (NO, iNOS) were suppressed in the LF group but enhanced by YPF supplementation. Microbial community analysis showed reduced diversity and altered composition in the LF group, with increased Proteobacteria and decreased Firmicutes, which were partially restored by YPF. Correlation analysis revealed that *Lactobacillus* and *Chitinibacter* play pivotal roles in regulating intestinal health. *Lactobacillus* exhibited a positive relationship with the intestinal PM and immune-related indicators, whereas *Chitinibacter* was negatively associated with these factors.

**Discussion:**

These results highlight the adverse impacts of a low-fishmeal diet on the intestinal health of M. rosenbergii and demonstrate the beneficial effects of YPF polysaccharides in alleviating these negative consequences through various mechanisms, including improved growth performance, enhanced antioxidant enzyme activities, restored intestinal ultrastructure, and modulated immune responses. The findings suggest that YPF supplementation could be a valuable strategy for mitigating the negative effects of low-fishmeal diets in aaquaculture.

## 1 Introduction

The surge in global raw material and labor costs has intensified inflation, a trend particularly evident in the aquaculture feed industry, highlighted by the sharp increase in fishmeal prices (1). As economic globalization advances, the demand for high-protein aquaculture feeds rich in fishmeal has risen steadily across countries, pushing up the market price of premium fishmeal. This has prompted an urgent need within the industry to seek economically viable alternative protein sources to partially replace the expensive fishmeal in aquaculture feeds. Plant proteins, known for their high protein content, cost-effectiveness, balanced amino acid composition, and high digestibility, have become ideal substitutes for fishmeal (2). Prudent replacement of fishmeal with plant proteins not only lowers feed costs but also preserves wild resources and reduces environmental impact (3).

However, the extensive replacement of fishmeal with plant proteins in aquaculture feeds may lead to adverse effects, including reduced digestibility, stunted growth, intestinal mucosal damage, and diminished antioxidant activity and immune competence in aquatic animals (4, 5). Moreover, excessive use of plant proteins may disrupt the balance of intestinal microbiota in aquatic organisms, impairing intestinal barrier function, reducing nutrient digestion and absorption, and inducing intestinal inflammation (6, 7). Studies have shown that the substitution of fishmeal with plant protein sources such as soybean meal results in a decrease in the relative abundance of beneficial intestinal bacteria and an increase in pathogenic bacteria in aquatic animals (8, 9). The peritrophic matrix (PM), a critical structure in the intestines of crustaceans, composed of chitin and proteins, plays a vital role in maintaining intestinal health and immune function (10). It serves not only as a physical barrier preventing direct contact of pathogens with intestinal cells but also regulates immune responses, promotes nutrient absorption, influences the balance of intestinal microbiota, and enhances the host’s defense against pathogens, collectively maintaining the intestinal health and overall immune status of crustaceans (11, 12). Previous research has indicated signs of PM dissolution in crustaceans such as *Eriocheir sinensis* under low-fishmeal diets (13). Therefore, identifying a feed additive that can protect the morphology of the PM and maintain intestinal health and microbiota balance is of paramount importance.

In recent years, to mitigate the adverse effects of plant protein substitution on aquatic animals, there has been a growing focus on using Chinese herbal polysaccharides as feed additives (14, 15). Notably, Yu Ping Feng (YPF) polysaccharide has been shown to exert a significant immunomodulatory effect, positively enhancing the immune function of aquatic animals (16). YPF is a traditional Chinese medicine compound consisting of Astragalus membranaceus, Atractylodes macrocephaia Koiz, and Saposhnikovia divaricata (17). The active components of these herbs, such as polysaccharides, flavonoids, and polyphenols, are particularly notable for their immunomodulatory effects, as demonstrated in numerous studies. For instance, flavonoids and plant polyphenols have been shown to enhance the activity of natural killer cells and improve immune response (18, 19). Polysaccharides, while known for their prebiotic properties, promote the growth of beneficial intestinal bacteria. As a mixture of these three herbs, YPF polysaccharide possesses a more diverse array of bioactive components compared to any single herb or combination of two herbs (20). In aquaculture, YPF polysaccharide not only exhibits superior growth-promoting effects but also improves intestinal function, enhances intestinal digestive enzyme activity and antioxidant capacity, and increases animal growth performance (16). In mammals, YPF polysaccharide has shown significant benefits in promoting the colonization of beneficial bacteria in the intestine and treating intestinal microbial dysbiosis caused by pathogens (21). However, research on the impact of YPF polysaccharide on PM formation and its role in maintaining intestinal health and microbiota balance remains insufficient.

*Macrobrachium rosenbergii*, a globally cultivated prawns species, holds significant economic value and serves as an important model for studying crustacean nutrition, metabolism, and immunology. The intestine plays a pivotal role in the nutritional absorption, immune defense, and overall health of crustaceans. This vital organ not only facilitates the efficient uptake of essential nutrients but also serves as a first line of defense against pathogenic invasions (22). Previous research has investigated the intestinal immune regulation and host health in *M. rosenbergii* (23), while recent studies have increasingly highlighted the role of gut microbiota in health and immune regulation among crustaceans (24). Developing feed additives for low-fishmeal diets in *M. rosenbergii* to enhance intestinal health is economically and scientifically significant. This study evaluates the potential of YPF polysaccharide to promote growth, protect intestinal morphology, and maintain health, thereby supporting sustainable aquaculture.

## 2 Materials and methods

### 2.1 Experimental animals and ethical statement

*M. rosenbergii* with an average body weight of 0.15 ± 0.01g were procured from the Freshwater Fisheries Research Center (FFRC) of the Chinese Academy of Fishery Science (CAFS). These prawns were temporarily housed for a period of seven days in three aerated tanks (dimensions: R × H, 1.0 m × 1.5 m) and were provided with commercial feed from Fuyuda Food Products Co. LTD, China, prior to the commencement of formal breeding experiments.

The experimental protocol adhered to the guidelines for scientific breeding and ethical use of animals, which were informed by our previously published work (1).

### 2.2 Experimental design and conditions

Based on our previous findings from growth and biochemical studies investigating the effects of YPF polysaccharide supplementation at concentrations ranging from 0 to 1000 mg/kg (**Supplementary Tables S1**, **S2**), we have formulated experimental diets. Formulating low fishmeal diets by replacing 40% of fishmeal with soybean meal. The experimental diets, as detailed in **Table 1**, comprise four distinct formulations: the normal fish meal diet (NF), the low fishmeal diet (LF), and the LF diet supplemented with 500 mg/kg (500P) and 1000 mg/kg YPF polysaccharide (1000P). Fish oil and soybean oil were included as lipid sources, and plant and animal meals were used as carbohydrate sources. Bentonite served as a feed binder, while squid paste acted as a feeding attractant. The actual concentrations of YPF polysaccharide were 500 and 1000 mg/kg, respectively. The ingredients were thoroughly mixed with an appropriate amount of water. The dough was then passed through a mincer, producing 1-mm-diameter strings. After drying in cool and shady conditions, the diets were broken up and stored in a refrigerator at -20°C until use.

**Table 1:** Ingredients and proximate chemical composition of the experimental diets.

Ingredients (%)	Groups			
	CT	LF	500P	1000P
Fish meal^a^	25.00	15.00	15.00	15.00
Soybean meal^b^	19.00	33.00	33.00	33.00
Rapeseed meal	15.50	15.50	15.50	15.50
Shrimp powder	4.00	4.00	4.00	4.00
Squid paste	3.00	3.00	3.00	3.00
α -starch	20.50	15.71	15.71	15.71
Fish oil:soybean oil (1:1)	2.00	2.70	2.70	2.70
Chellocken meal	3.00	3.00	3.00	3.00
Poultry by-product powder	2.00	2.00	2.00	2.00
Soybean phospholipids	1.00	1.00	1.00	1.00
Cholesterol	0.50	0.50	0.50	0.50
Ca(H_2_PO_4_)_2_	2.00	2.00	2.00	2.00
Choline chloride	1.00	1.00	1.00	1.00
Remix^c^	1.00	1.00	1.00	1.00
Bentonite	0.990	0.990	0.940	0.890
Ecdysone (10%)	0.01	0.01	0.01	0.01
Yupingfeng polysaccharide	0.00	0.00	0.05	0.1
Proximate Composition				
Crude protein	38.36	38.28	38.28	38.28
Ether extract	8.24	8.23	8.23	8.23
Gross energy	17.89	17.70	17.70	17.70

^a^Crude protein of fish meal: 65.57%; ^b^Crude protein of cottonseed protein concentrate: 65%; ^c^ Vitamins and mineral premix (IU, g or mg kg-1 of diet): vitamin A, 25000 IU; vitamin D3, 20000 IU; vitamin E, 0.2 g; vitamin K3, 0.02 g; ammonium sulfate, 0.04 g; riboflavin, 0.05g; calcium pantothenate, 0.01g; pyridoxine hydrochloride, 0.04g; vitamin B12, 0.2 mg; biotin, 6mg; folic acid, 20 mg; nicotinic acid, 200 mg; inositol, 1g; vitamin C, 2g; choline, 2g; calcium dihydrogen phosphate, 20g; sodium chloride, 2.6g; potassium chloride, 5 g; magnesium sulfate, 2000mg; ferrous sulfate, 900 mg; zinc sulfate, 60 mg; copper sulfate, 20 mg; magnesium sulfate, 30 mg; sodium selenate, 20 mg; cobalt chloride, 50mg; potassium iodide, 4mg.

In addition, a total of 480 prawns with an initial body weight of 0.15 ± 0.01 g were distributed into twelve tanks (dimensions: 1.0 m × 1.5 m), with 40 prawns per tank. Twelve tanks were randomly assigned to three experimental diet groups and one control group. Prawns were provided feed three times daily at 8:00, 13:00, and 18:00 until apparent satiation. Residual feed was collected using 80 mesh net bags one hour post-feeding.

### 2.3 Growth evaluation

Post the 8-week feeding trial, the survival rate, final body weights, and body lengths of prawns within each group were recorded. Concurrently, the tail muscle of the prawns was isolated and weighed. The weight gain rate, specific growth rate, coefficient of fatness, and flesh rate were calculated using the following methodologies:

Weight gain rate (WGR, %) = (Body weight (g) − Initial weight (g))/initial weight × 100

Specific growth rate (SGR, %/day) = (Ln Body weight − Ln initial weight) × 100/days

Feed conversion ratio (FCR) = Dry feed intake (g)/weight gain (g)

Coefficient of condition (CF) = Body weight (g)/Body length (cm)^3^

Flesh rate (FR) = (Muscle weight (g)/Body weight) × 100

### 2.4 Samples collection

Following an 8-week feeding trial, 6 prawns were randomly selected from each tanks (18 per group) for further analysis. Initially, Elsevier’s solution, comprising 13.2g/L trisodium citrate, 4.8g/L citric acid, and 14.7g/L glucose, was employed as an anticoagulant to facilitate the collection of hemolymph. Adhering to the methodology outlined by Rodriguez et al., a 1ml syringe was de-aired, filled with 200μl of anticoagulant, and used to extract hemolymph from the thoracic region of the prawns, maintaining a 1:1 ratio of hemolymph to anticoagulant. The resultant mixtures were then centrifuged at 4000 rpm for 10 minutes at 4°C to separate the hemolymph from blood cells, with the supernatant from three prawns in each replicate being transferred to a 1.5 ml centrifuge tube.

Subsequently, the prawns were dissected to procure midgut and chyme samples. The intestines from three prawns were randomly mixed in each replicate and placed into a 2 ml cryogenic vial, with the chyme being similarly stored. These cryogenic vials were subsequently snap-frozen in liquid nitrogen and stored at -80°C for subsequent analysis. Additionally, three intestines from each diet were randomly collected, fixed in a solution of 4% paraformaldehyde and 2.5% glutaraldehyde (supplied by Macklin Biochemical Co., Ltd, Shanghai, China) for histological and transmission electron microscopy (H&E and TEM) analysis, respectively. The remaining prawns samples were immediately stored in a freezer at -80°C to serve as a reserve for supplementing any insufficient test samples.

### 2.5 Enzyme activity analysis

In accordance with the methodologies delineated in our prior research (25), the activities of superoxide dismutase (SOD), catalase (CAT), and glutathione peroxidase (GSH-Px) in hemolymph were quantified using commercial bioengineering kits from the Nanjing Jiancheng Institute. These enzyme activities were assessed via a Spectra Max Plus spectrophotometer (Molecular Devices, Menlo Park, CA, USA), with measurements taken at wavelengths of 450 nm, 550 nm, 420 nm, and 530 nm, respectively. The concentration of malondialdehyde (MDA) in the hemolymph was determined through the thiobarbituric acid (TBA) method, with quantification performed at a wavelength of 532 nm (26).

For the determination of intestinal enzyme activities, including SOD, CAT, GSH-Px, inducible nitric oxide synthase (iNOS), nitric oxide (NO), and lysozyme (LZM), as well as MDA and lipopolysaccharide (LPS), a pretreatment protocol is necessary. This protocol is adapted from the previously published work by Liu et al. (27). Following the extraction of the supernatant for subsequent enzyme activity analysis, the parameters for intestinal SOD, CAT, GSH-Px, MDA, NO, iNOS, LZM, and LPS were measured analogously to those of the hemolymph. The activities of intestinal NO and iNOS were analyzed using commercial bioengineering kits from the Nanjing Jiancheng Institute, with measurements taken at wavelengths of 550 nm and 530 nm, respectively. The analyses of LZM and LPS were conducted using commercial bioengineering kits from Beijing Solarbio Co., Ltd., with measurements performed at wavelengths of 530 nm and 420 nm, respectively.

### 2.6 H&E, TUNEL and Oil Red O stainings

Fixed intestine samples, initially preserved in a 4% paraformaldehyde buffer, were subsequently embedded in optimum cutting temperature (OCT) compound and stored at -80°C. Following the protocol detailed in our published work (28), Hematoxylin-eosin (H&E) staining was performed on the intestine samples, and the resulting microstructures were imaged using a Leica DM1000 optical microscope (Wetzlar, Germany). Intestinal tissue, previously fixed in a 2.5% glutaraldehyde solution, was subjected to preprocessing and sectioning in accordance with the methodology described by Xie et al. (29). The cellular ultrastructure was then examined using a transmission electron microscope (Hitachi HT7700, Japan).

### 2.7 Quantitative real-time RT-PCR validation

According to the fragments obtained from the earliest intestine transcriptome determined initially in our laboratory and the ORF intercepted, designing the gene primer by Primer 5.0 for qRT-PCR analysis (**Table 2**). Choosing β-actin serves as an internal reference due to its stable expression. Jiangsu Gencefe Biotechnology Co., LTD synthesized PCR primers.

**Table 2:** PCR primer sequence of gene of *M. rosenbergii*.

Gene	Primer sequences (5′-3′)	Bp	Size	E (%)	Sequence source
*Toll*	(F) TTCGTGACTTGTCGGCTCTC (R) GCAGTTGTTGAAGGCATCGG	20	194	103.21	KX610955.1
*Dorsal*	(F) TCAGTAGCGACACCATGCAG (R) CGAGCCTTCGAGGAACACTT	20	280	94.52	KX219631.1
*IMD*	(F) CGACCACATTCTCCTCCTCCC (R) TTCAGTGCATCCACGTCCCTC	21	235	94.59	OR602697
*Relish*	(F) GATGAGCCTTCAGTGCCAGA (R) CCAGGTGACGCCATGTATCA	20	140	106.32	KR827675.1
*IkBα*	(F)AATCATACCGGAAGGACGGCGTTA (R)TCACGGGTCTGGTTAATTGGGTCA	24	280	100.69	HQ668091.1
*GS*	(F) AGCCTGCCTCTACACTGGTA (R) TGACGCCGAAATCTTCAGCT	20	163	98.37	Imaizumi et al., 2023
*CHS*	(F) ACCCATTGGTTTGGTGTTCG (R) TATGCGAAATGGTGCCGAAG	20	109	99.12	Lu et al., 2019
*EcPT*	(F) TTGTGACTGGCCACAGAACG (R) GGCGAGACTGCTTGTCGAAT	20	234	97.84	Wang et al., 2013
*β-actin*	(F) TCCGTAAGGACCTGTATGCC (R) TCGGGAGGTGCGATGATTTT	20	126	90.08	KX610955.1

E, reaction efficiencies.

The total RNA of the intestine in the four experimental groups (three intestine mixtures per replicate) was extracted with RNAiso Plus (TaKaRa, Japan). Then, the concentration was determined with Nanodrop 2000 (Thermo Fisher Scientific, Waltham, Massachusetts, USA). The RNA concentration of each sample was diluted to 400 ng/mL. According to the manufacturer’s directions, First-strand cDNA was generated from 400 ng DNase-treated RNA using an HiScript III 1st Strand cDNA Synthesis Kit (Vazyme Biotech Co., Ltd., Nanjing, China). Use the Two-Step SYBR^®^ Prime Script^®^ Plus RT-PCR Kit (TaKaRa, Japan) for the quantitative analysis of 2 µg of total RNA. The total system of sample loading was 20 µL. Based on Sun et al. (28), the PCR reaction conditions were as follows: 95°C for 30 s, followed by 40 cycles of 95°C for 5 s and 60°C for 30 s. The specificity of the primers was evaluated by melting curves and electrophoresis. The Bio-Rad CFX96 (Bio-Rad Laboratories, Inc., Hercules, USA) real-time PCR system was employed for real-time quantitative reverse transcription PCR (qRT-PCR). The 2−^ΔΔCT^ method was utilized to calculate the relative gene expression (30).

### 2.8 Bacterial 16S rRNA gene amplification, cDNA library construction and sequencing

To characterize the diversity and structure of microbial communities, the 16S rDNA V3-V4 region of the ribosomal RNA gene was amplified via PCR (95°C for 2 min, followed by 27 cycles at 98°C for 10 s, 62°C for 30 s, and 68°C for 30 s, with a final extension at 68°C for 10 min) using the primers 338F: ACTCCTACGGGAGGCAGCAG and 806R: GGACTACHVGGGTWTCTAAT, where the barcode is an eight-base sequence unique to each sample. PCR reactions were conducted in triplicate 50 μL mixtures, following the methodology of Xue et al. (31). All PCR products were extracted from 2% agarose gels and purified using the Merck DNA Gel Extraction Kit (Merck Sigma-Aldrich, Darmstadt, Germany) according to the manufacturer’s instructions, and quantified using the Bio-Rad CFX96 Real-Time PCR System (Bio-Rad Laboratories, Inc., Hercules, USA). Purified PCR amplicons were pooled in equimolar concentrations and subjected to paired-end sequencing (2 × 250) on an Illumina platform, adhering to standard protocols provided by the manufacturer.

Following these steps, clean reads were obtained. Barcode and linker sequences were removed, and paired-end reads were combined into longer fragments. Reads with average quality scores below 20 and lengths less than 100 bp were excluded. Sequences with mismatched primer sequences or ambiguous bases (Ns) exceeding 5% were removed from downstream analyses. Unassembled reads were discarded. Spliced paired-end sequences were generated using FLASH. Chimeric sequences were eliminated using VSEARCH. Sequences were then clustered into operational taxonomic units (OTUs) at a 97% sequence similarity threshold using UPARSE (version 7.1), and taxonomic classifications were annotated in the RDP database (1).

Microbial variation was assessed through a multifaceted analytical approach. Principal coordinate analysis (PCoA) and heatmaps depicting the composition of operational taxonomic units (OTUs) were generated using R software. The Stamp tool, accessible through the Tutools platform (http://www.cloudtutu.com/), was employed to discern differences in microbial abundance at the genus level among the three dietary treatments. The Kruskal-Wallis test was utilized to evaluate differences across all samples, and KEGG histograms were produced using Prism software version 8.0. Correlation analysis between intestinal microbes and metabolites was performed with R software, resulting in the generation of network diagrams. These network diagrams were subsequently refined using the Gephi software.

### 2.9 Statistical analysis

All experimental results were presented as mean ± standard error of the mean (S.E.M.). The Duncan multiple range test was employed to assess the differences among the four groups. Significant differences (*p* < 0.05) among different groups are indicated by different lowercase letters on the histogram. The statistical analysis was conducted using SPSS software version 16.0 (SPSS Inc., Michigan Avenue, Chicago, IL, U.S.A.). Pearson correlation analysis was utilized to examine the relationship between two variables that met the criteria for normality, with the following significance indicators: a single asterisk (*) denoted a significant difference (*p* < 0.05), and a double asterisk (**) signified a highly significant difference (*p* < 0.01).

## 3 Result

### 3.1 Growth performance

**Figure 1:**
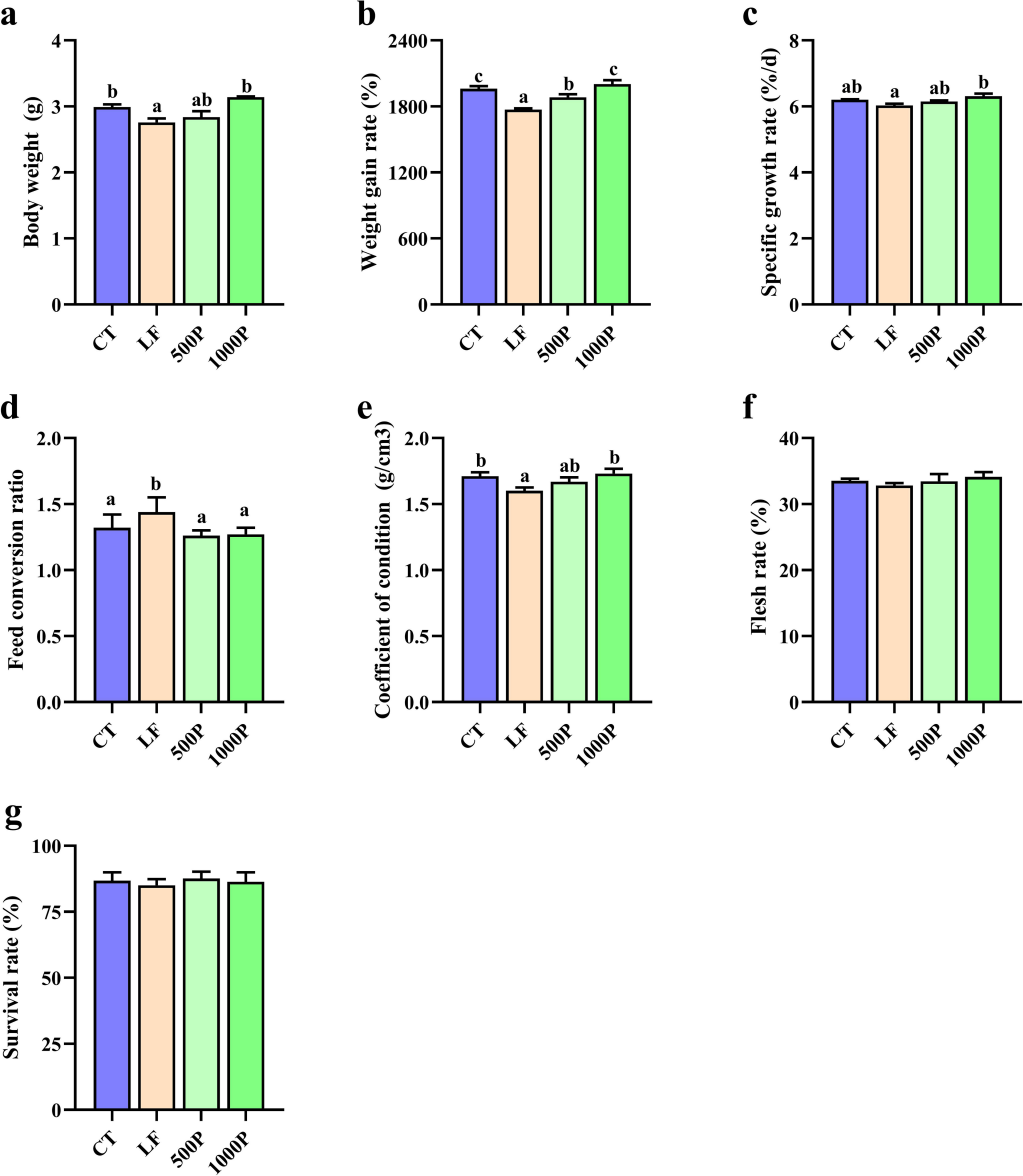
Effects of four diet on growth evaluation of *M. rosenbergii*. The six indicators body weight **(A)**, weight gain rate **(B)**, specific growth rate **(C)**, Feed conversion ratio **(D)**, coefficient of condition **(E)**, flesh rate **(F)** and survival rate **(G)** were compared in each group. Significant differences between the four groups are indicated by different lowercase letters (*p* < 0.05).

As shown in **Figure 1**, BW (body weight), WGR (weight gain rate), and CF (condition factor) in the LF group were significantly lower than in the CT group (*p* < 0.05). The inclusion of YPF polysaccharides in LF diets resulted in increased levels of BW, WGR, SGR, and CF (*p* < 0.05). Particularly significant improvements were observed in the 1000P group, as the WGR in the 1000P group was significantly higher than that in the 500P group (*p* < 0.05). Conversely, the FCR in the LF group was significantly higher than in the other three groups (*p* < 0.05). Lastly, there were no statistically significant differences in FR (flesh rate) and SR (survival rate) among the four groups (*p* > 0.05).

### 3.2 Antioxidant enzyme activity in hemolymph and intestine

**Figure 2:**
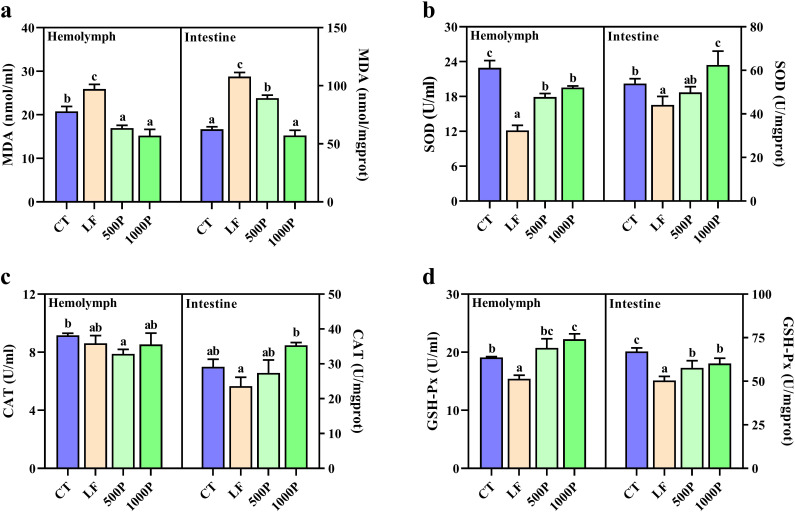
Related antioxidant enzyme activities in hemolymph and intestines of *M. rosenbergii* during the experimental period. **(A)** MDA, malondialdehyde. **(B)** SOD, superoxide dismutase. **(C)** CAT, catalase. **(D)** GSH-Px, glutathione peroxidase. Significant differences between the four groups are indicated by different lowercase letters (*p* < 0.05).

Four dietary groups exhibited significantly different levels of antioxidant enzyme activity in the hemolymph and intestine (**Figure 2**). Both in the hemolymph and intestine, compared to the CT group, the LF group showed significantly reduced levels of SOD and GSH-PX, MDA content was significantly elevated (*p* < 0.05). In addition, the 500P and 1000P groups, which were supplemented with YPF polysaccharides, exhibited significantly elevated levels of SOD and GSH-PX and significantly reduced levels of MDA compared to the LF group (*p* < 0.05). Moreover, the 1000P group exhibited a more pronounced effect. Notably, the CAT levels in the hemolymph of the LF group did not exhibit significant differences compared to the other three groups (*p* > 0.05); however, mirroring the results observed for SOD in the gut, the addition of 1000 mg/kg YPF polysaccharides to the LF group led to CAT levels that were higher than those in the control group.

### 3.3 Ultrastructural observation of the intestine

**Figure 3:**
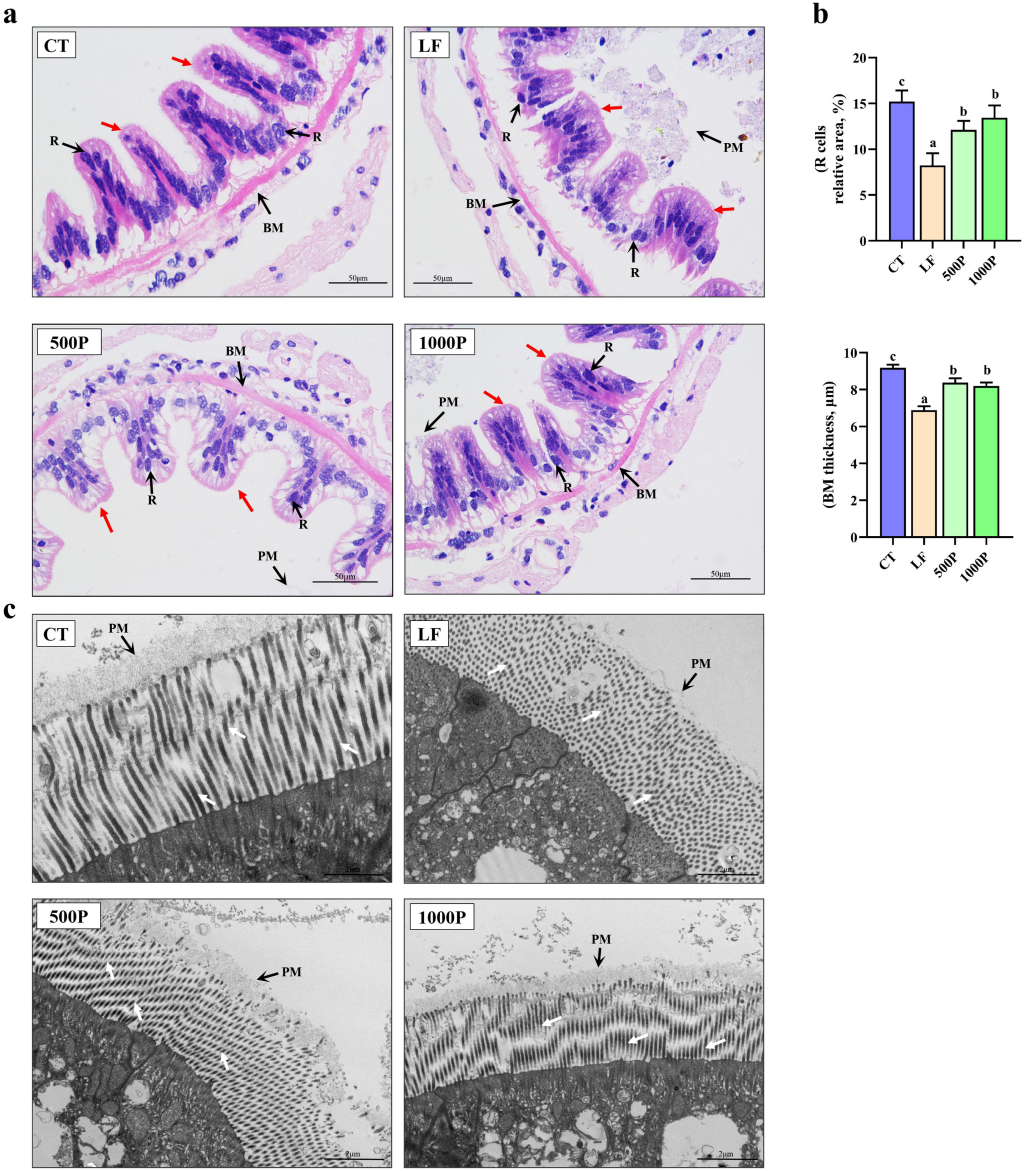
Intestinal histological structure alterations of *M. rosenbergii*. **(A)** Microscopy of H & E stained intestinal structures. PM, Peritrophic Matrix; R, Regenerative Cell; BM, Basement Membrane. **(B)** The relative R cell area and BM thickness. **(C)** Transmission electron microscopy of intestinal structure. Red arrows represent intestinal villus, white arrows represent intestinal microvilli. Significant differences between the four groups are indicated by different lowercase letters (*p* < 0.05).

As shown in **Figure 3A**, intestinal villi of the control group are neatly arranged and closely connected to the basement membrane (BM), with R cells evenly and densely distributed on the intestinal villi. In contrast, the LF group exhibited detachment of intestinal villi from the BM, accompanied by a visible reduction in R cells, and the shedding of the PM was also observed. However, the addition of YPF polysaccharide to the LF group alleviated the detachment of the BM and the reduction of R cells, and the shedding of the PM also seemed to be improved. Statistically, the BM thickness and the number of R cells in the LF group were significantly lower compared to the other three groups (*p* < 0.05); Furthermore, these two indicators in the YPF polysaccharide-supplemented group were significantly lower than those in the control group (*p* < 0.05, **Figure 3B**).

Further observation of the structure of intestinal microvilli using transmission electron microscopy revealed that the LF group exhibited extensive shedding, necrosis, and scattered distribution of microvilli, with no perisoteal membrane observed to be tightly attached to the microvilli compared to the CT group (**Figure 3C**). However, the addition of YPF polysaccharide improved the damaged structure of intestinal microvilli and the perisoteal membrane, especially after the addition of 1000 mg/kg of YPF polysaccharide, where the microvilli on the surface of the intestinal epithelial cells were evenly distributed and neatly arranged, and the perisoteal membrane was tightly attached to the microvilli.

### 3.4 Determination of intestinal peritrophic matrix related indicators

**Figure 4:**
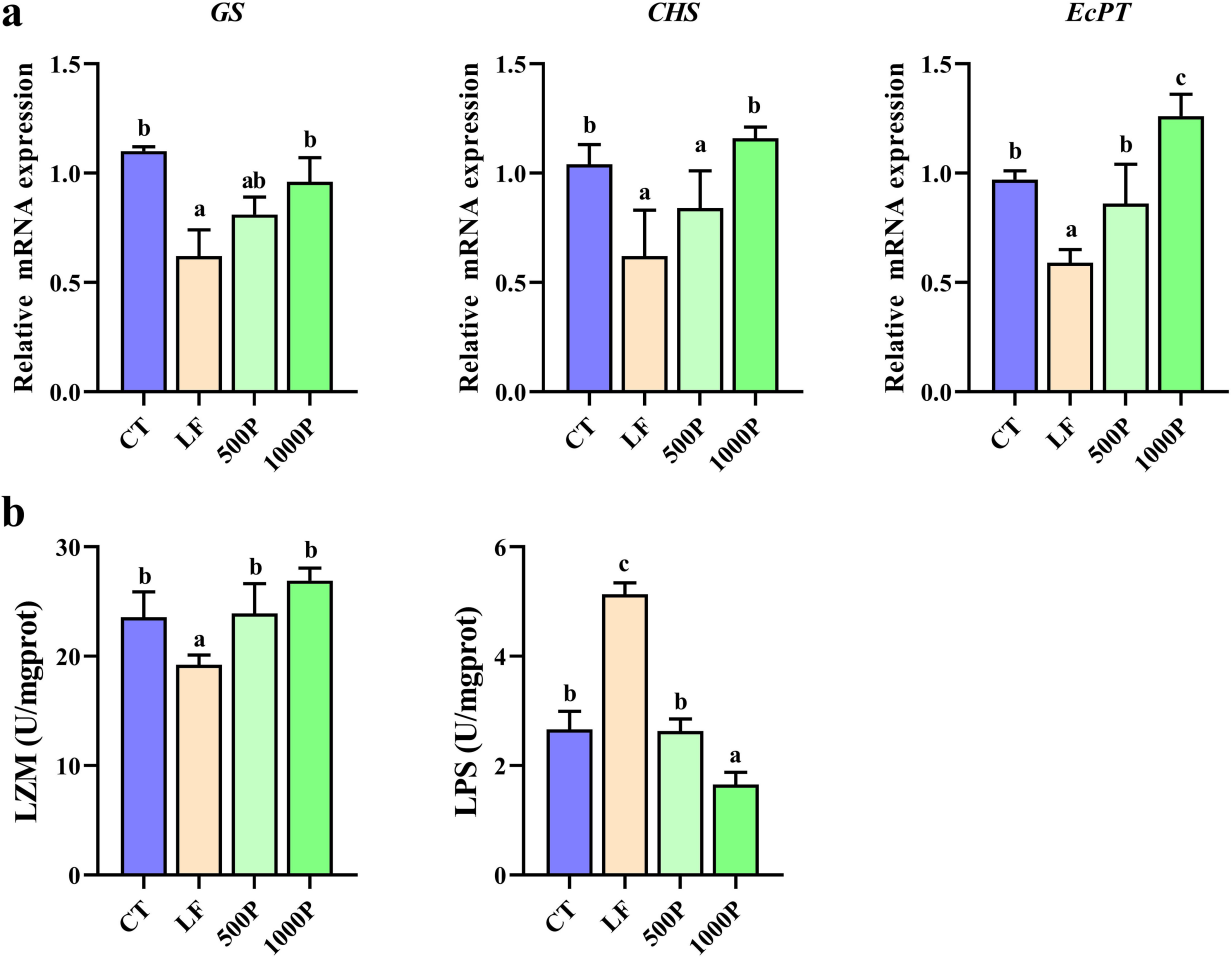
Changes in peritrophic matrix-related genes and enzymes in *M. rosenbergii*. **(A)** Gene mRNA expression of glutamine synthetase (GS), chitin synthase (CHS) and peritrophin-like protein (EcPT). **(B)** Related antioxidant enzyme activities of lysozyme (LZM) and lipopolysaccharide (LPS). Significant differences between the four groups are indicated by different lowercase letters (*p* < 0.05).

As shown in **Figure 4A**, significant alterations in the expression levels of several genes involved in peritrophic matrix formation were evident. The expression level of the *GS*, *CHS* and *EcPT* gene in the LF group was significantly decreased compared to the control group, and it was also markedly lower when compared to the 1000P group (*p* < 0.05). Additionally, the expression levels of *EcPT* in the 1000P group exhibited a significant increase compared to those in the control. It is noteworthy that the trends in the levels of LZM and LPS also exhibited opposite patterns (**Figure 4B**). The LZM level in the LF group was significantly lower than in the other three groups (*p* < 0.05), whereas the LPS level was significantly elevated (*p* < 0.05).

### 3.5 Determination of intestinal non-specific immunity related indicators

**Figure 5:**
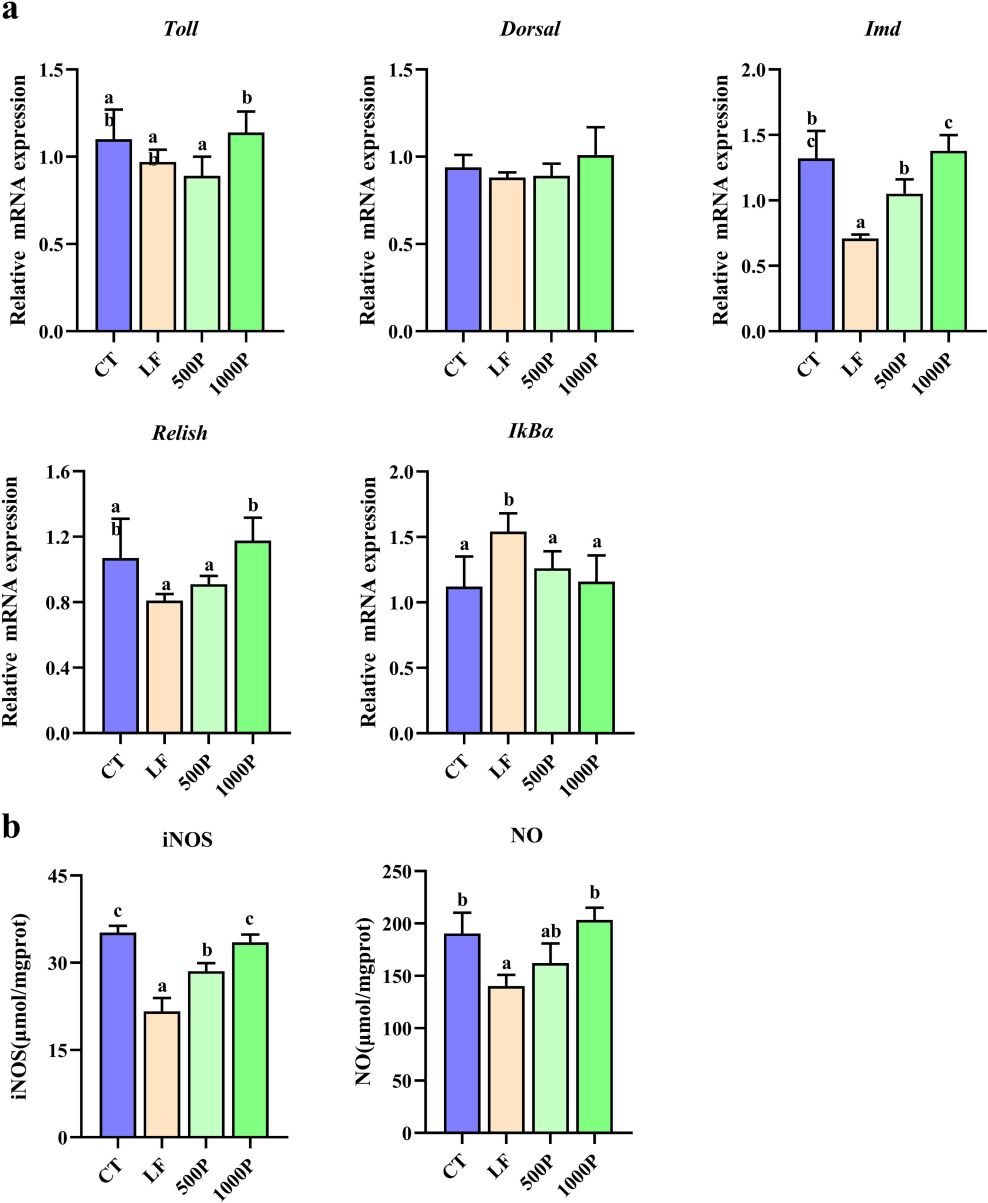
Changes in non-specific immunity related genes and enzymes in *M. rosenbergii*. **(A)** Gene mRNA expression. **(B)** Related antioxidant enzyme activities of inducible nitric oxide synthase (iNOS) and nitric oxide (NO). Significant differences between the four groups are indicated by different lowercase letters (*p* < 0.05).

As depicted in **Figure 5A**, both the LF group and the addition of YPF polysaccharide at 1000 mg/kg exerted significant effects on the relative expression levels of genes associated with non-specific immunity in *M. rosenbergii*. The expression levels of *IMD* and *Relish* mRNA in the LF group were significantly lower than those in the control group (*p* < 0.05); however, after the addition of 1000 mg/kg YPF polysaccharide, the expression levels of *IMD* and *Relish* mRNA significantly increased (*p* < 0.05). For *IκBα*, both the control group and the YPF polysaccharide-supplemented group exhibited significantly lower expression levels compared to the LF group (*p* < 0.05). Additionally, there were no significant differences in the expression levels of *Toll* and *Dorsal* mRNA among four groups (*p* > 0.05).

**Figure 5B** illustrates the activity levels of two enzymes associated with non-specific immune responses in prawns. It was observed that the levels of NO and iNOS in the LF group were significantly reduced compared to the control (*p* < 0.05). However, after the addition of 1000 mg/kg YPF polysaccharide, the activity levels of these two enzymes significantly increased in comparison to the LF group (*p* < 0.05), reaching levels comparable to those of the control.

### 3.6 Alterations in the microecological structure in the intestine

**Figure 6:**
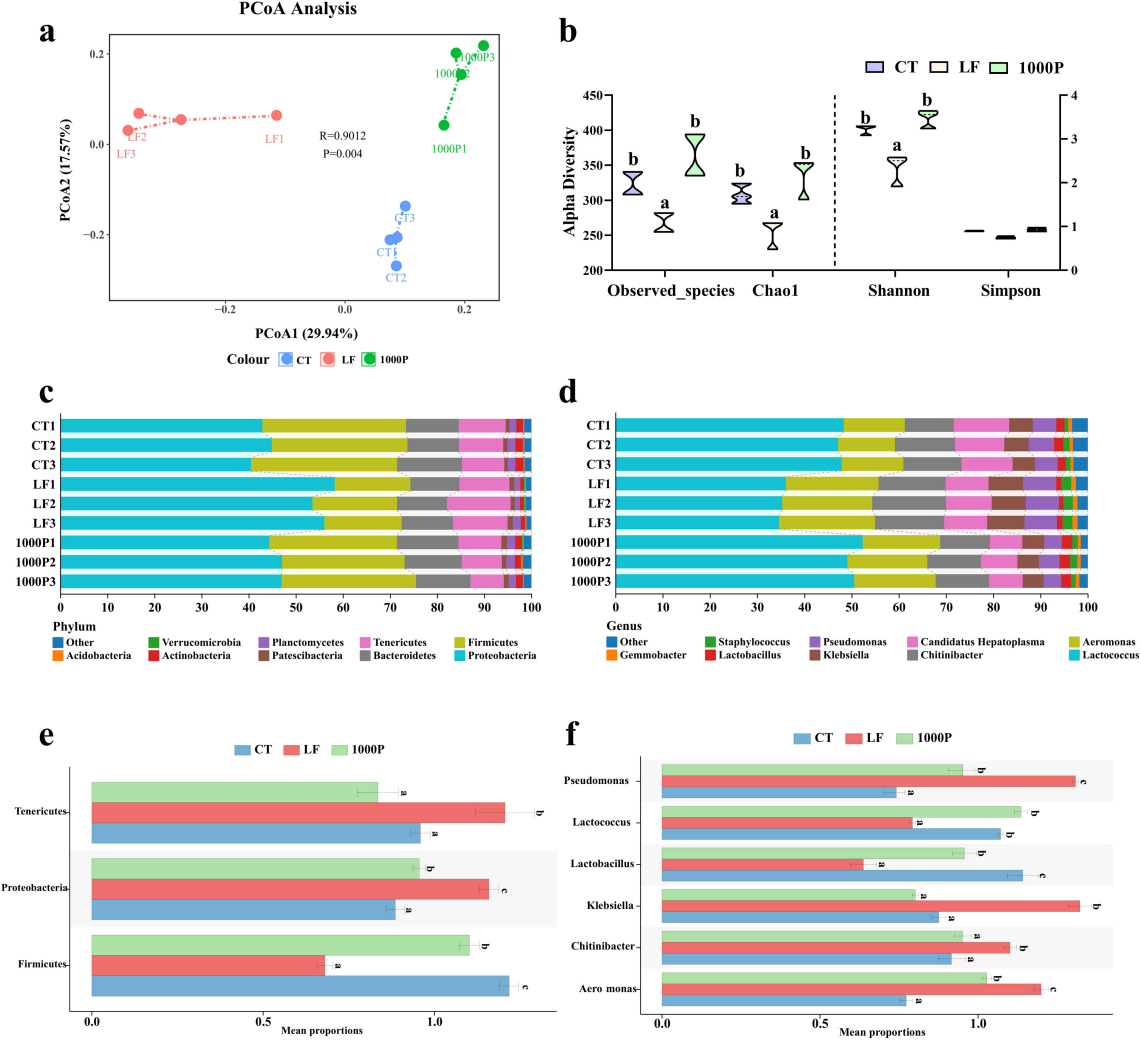
Effects of three diets on the intestinal microflora. **(A)** Principal coordinate analysis of community. **(B)** Alpha diversity indices. **(C, D)** Histogram analysis of microbial taxonomic composition at phylum and genus levels in the top ten, respectively. **(E, F)** Microbial comparation analysis on phylum and genus level. Significant differences between the four groups are indicated by different lowercase letters (*p* < 0.05).

Due to previous results indicating that the addition of 1000 mg/kg YPF polysaccharide had a more pronounced effect compared to the addition of 500 mg/kg, the structural changes in the intestinal microbiota were only compared among the 1000P group, the control group, and the LF group (**Figure 6**). The principal coordinate analysis (PCoA) results showed that the microbial composition in the LF group was evidently different from the control and 1000P groups (**Figure 6A**). Compared to the LF group, the microbial community in the CT and 1000P groups exhibited shorter dispersal distances. Meanwhile, Observed_species, Goods_coverage, Chao1, Shannon, and Simpson indices were calculated based on the OTUs to evaluate each group’s microbial community diversity (**Figure 6B**). The Observed_species, Chao1, and Shannon in the LF group were significantly lower than in the CT and 1000P groups (*p* < 0.05). In addition, the Simpson results showed that there were no statistically significant differences among the three groups (*p* > 0.05).

The analysis of microbial composition revealed that at the phylum level (**Figure 6C**), Proteobacteria, Firmicutes, Bacteroidetes, and Tenericutes were the predominant bacteria in the intestinal microecology of prawns across the three groups. Specifically, when examining the microbial composition of each group (**Figure 6E**), it was observed that in the LF group, the levels of Proteobacteria and Tenericutes were significantly higher than those in the control group and the 1000P group, whereas the level of Firmicutes was significantly lower than in these two groups (*p* < 0.05). Additionally, compared to the control group, the level of Firmicutes in the 1000P group was significantly increased, while the level of Proteobacteria was significantly decreased (*p* < 0.05). At the genus level shown in **Figure 6D**, *Lactococcus*, *Aeromonas*, *Chitinibacter*, *Candidatus Hepatoplasma*, *Klebsiella*, *Pseudomonas*, and *Lactobacillus* were the dominant bacterial genera across the three groups. Comparing the levels of each genus within each group (**Figure 6F**), it was found that compared to the CT group and the 1000P group, the levels of *Aeromonas*, *Klebsiella*, and *Pseudomonas* were significantly increased in the LF group, while the levels of *Lactococcus* and *Lactobacillus* were significantly decreased (*p* < 0.05). Notably, the level of *Lactobacillus* in the 1000P group was significantly higher than in the control group, whereas the results for *Pseudomonas* and *Candidatus Hepatoplasma* were opposite (*p* < 0.05).

### 3.7 Enrichment function of intestinal microorganisms

**Figure 7:**
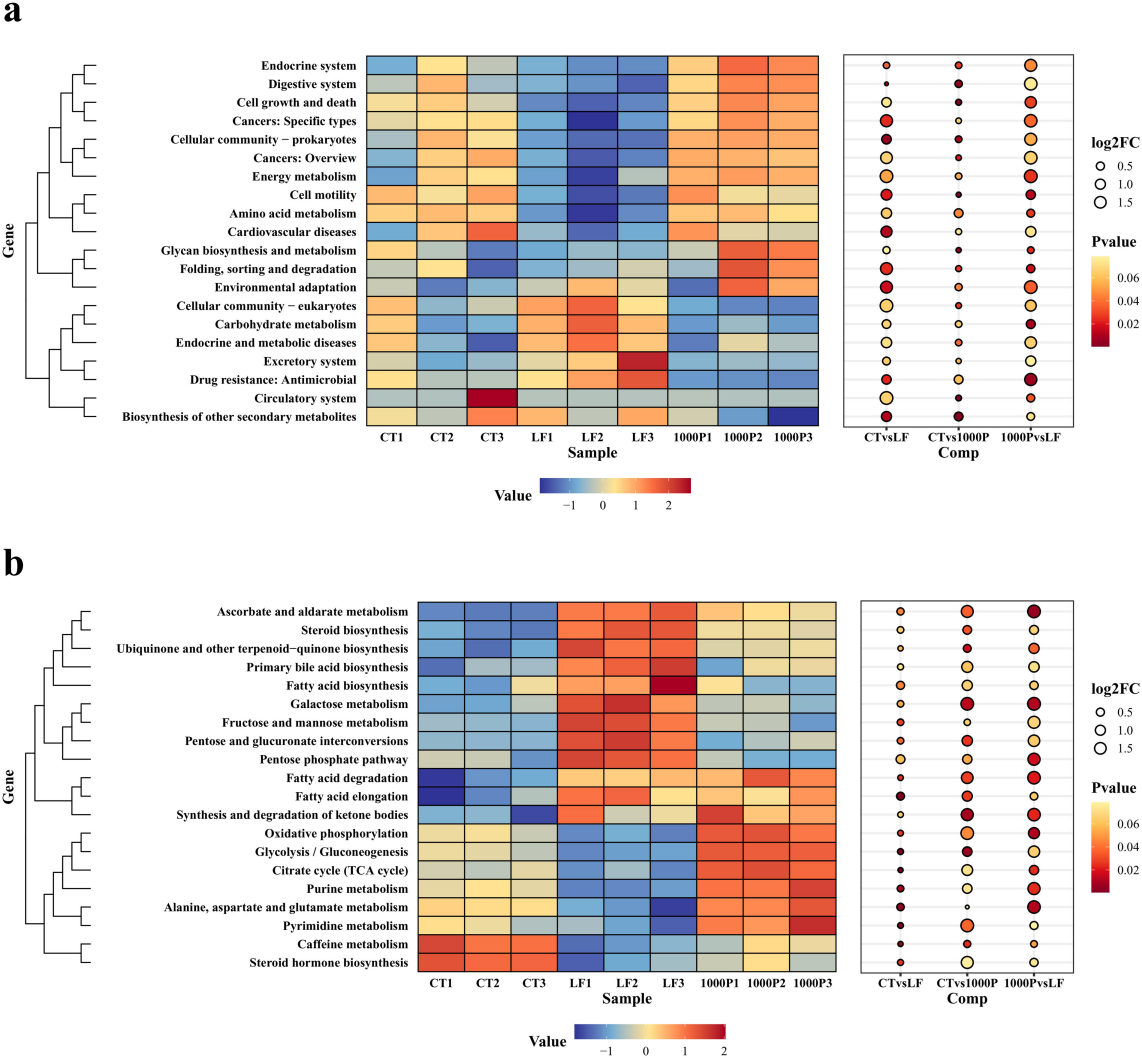
Effect of three diets on intestinal microbial functions. **(A)** Secondary functional annotation; **(B)** Tertiary functional annotation; red color represents upregulated function, and bule color represents downregulated function.

The prediction of the function of intestinal microorganisms in different diets is shown in **Figure 7**. The secondary function prediction (**Figure 7A**) showed that the LF group decreased the abundance of Cancers: Specific types, Cellular community-prokaryotes, Amino acid metabolism, and Cell motility pathways compared to the other two groups, and increased the abundance of Cellular community-eukaryotes, Carbohydrate metabolism, and Endocrine and metabolic diseases pathways (*p* < 0.05).

In addition, in tertiary function prediction (**Figure 7B**), the abundance of Galactose metabolism, Fructose and mannose metabolism, and Pentose and glucuronate interconversions in the LF group was lower than in the CT and 1000P groups. It’s worth noting that the 1000P group predicted a higher abundance of the Synthesis and degradation of ketone bodies, Oxidative phosphorylation, Glycolysis/Gluconeogenesis, Citrate cycle (TCA cycle), and Purine metabolism pathways than in the LF group, even in the CT group (*p* < 0.05).

### 3.8 Correlation analysis

**Figure 8:**
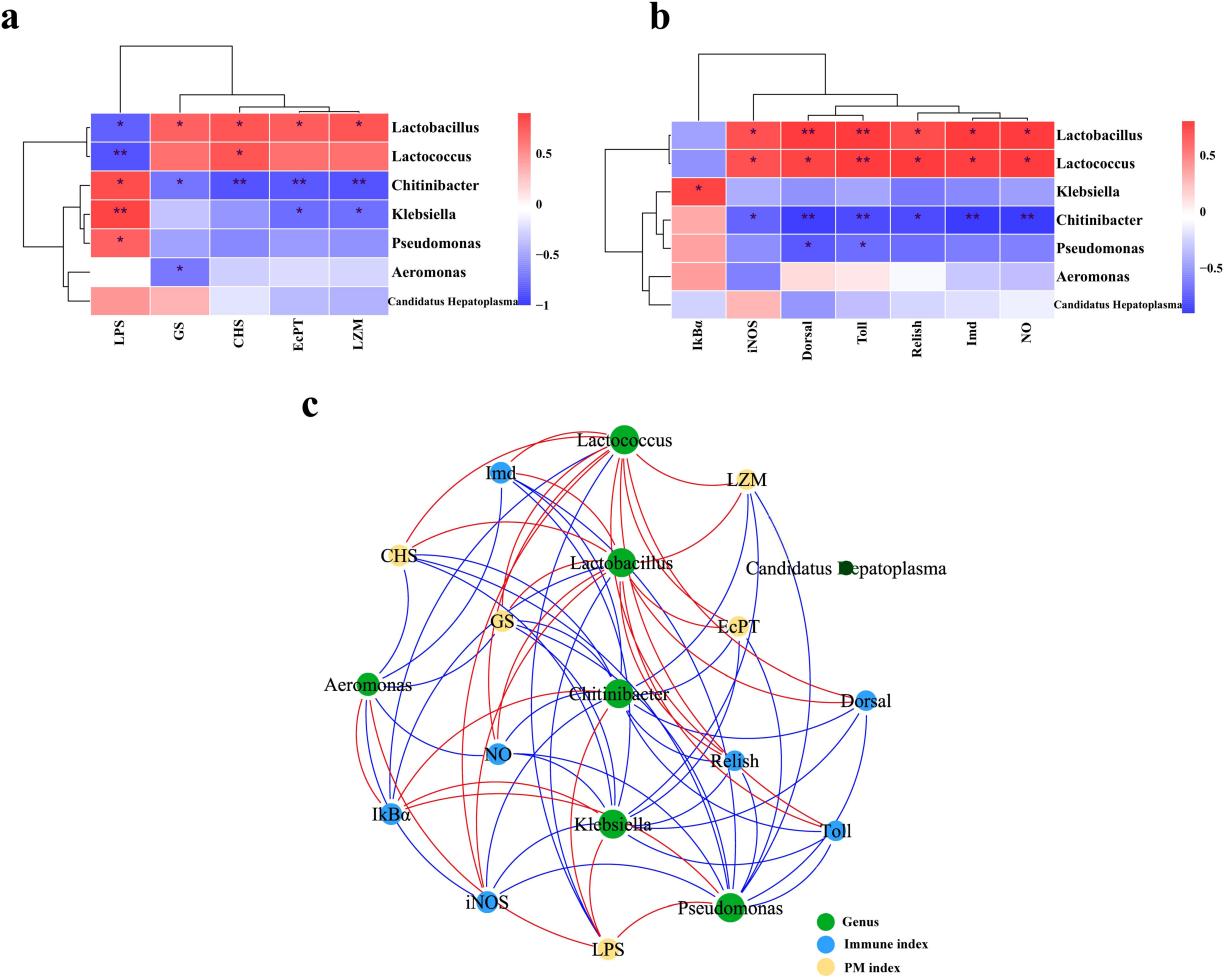
Correlation analysis. Correlation analysis between typical microbial genera and peritrophic matrix (PM) indices **(A)** and intestinal immune indices **(B)**, respectively. Red indicates positive correlation, while blue indicates negative correlation. The intensity of the color reflects the strength of the correlation. *p < 0.05, **p < 0.01. **(C)** Network diagram depicting the Spearman’s correlation of microbial genera with intestinal molecular indicators. A red line signifies positive correlation, a blue line signifies negative correlation, and the width of the line corresponds to the magnitude of the correlation.

**Figures 8A, B** presented the significance of correlation between seven intestinal genera with differentiated abundance and intestinal health indicators through Pearson’s correlation analysis. The comparative analysis of the correlation between intestinal microbiota and PM-associated molecular indicators (*GS*, *CHS*, *EcPT*, and LZM) revealed that *Lactobacillus* exhibited a significant positive correlation with these indicators, whereas *Chitinibacter* demonstrated a significant negative correlation (*p* < 0.05). Regarding LPS, *Lactobacillus* and *Chitinibacter* demonstrated a correlation trend that was contrary to LZM observations: *Lactobacillus* exhibited a negative correlation with LPS, while *Chitinibacter* showed a positive correlation (*p* < 0.05). Additionally, *Lactococcus* and *Lactobacillus* showed a significant positive correlation with the majority of intestinal immune indicators measured in this study: iNOS, NO, *Toll*, *Dorsal*, *Imd*, and *Relish* (*p* < 0.05); however, the results for *Chitinibacter* diverged, exhibiting an opposite trend. In the interpretation of the correlation network diagram (**Figure 8C**), it was observed that the genera Lactobacillus, *Chitinibacter*, and *Klebsiella* occupy central positions within the network and exhibit correlations with a majority of intestinal molecular indicators.

## 4 Discussion

In animal nutrition, growth performance serves as the most fundamental phenotypic indicator for assessing the impact of feed components on animal health (24). This study revealed that a low-fishmeal (LF) diet negatively affected the growth performance of *M. rosenbergii*, evidenced by significant reductions in BW, WGR, and CF. Such negative growth outcomes signify compromised health and immune function (32), although survival rates remained unaffected. Previous research has shown that prolonged consumption of a LF diet can lead to reduced growth performance and concurrent oxidative stress (33). Further studies have identified oxidative stress induced by a LF diet as a critical factor in diminished growth performance (1). To substantiate this, our study measured four oxidative stress-related indicators, including MDA, a marker of lipid peroxidation reflecting cellular oxidative damage (34), and three antioxidant enzymes (SOD, CAT, and GSH-Px) known to mitigate oxidative damage in crustaceans by eliminating excess free radicals (35). The elevated MDA levels and decreased antioxidant enzyme activities in the hemolymph of the LF group confirmed the anticipated oxidative stress damage.

To mitigate the growth and antioxidant deficits induced by the LF diet, YPF Polysaccharide was incorporated into the diet, leveraging its known antioxidant properties (16). YPF, derived from a traditional Chinese medicinal herb, is believed to enhance its bioactive potential due to the higher concentration of polysaccharides compared to the raw herb (36). In aquaculture, YPF has been used as a feed additive for grass carp, enhancing antioxidant enzyme activities and antioxidant capacity (37). Our study found that YPF supplementation improved oxidative stress damage by boosting antioxidant enzyme activities, providing evidence for the enhanced growth performance observed.

The relationship between oxidative stress and intestinal health has been a focal point in animal nutrition, given the intestinal critical role in nutrient digestion, absorption, and as the first line of defense against external pathogens (38). Our analysis of intestinal tissue antioxidant enzyme activities mirrored the findings in hemolymph, indicating oxidative stress damage induced by the LF diet. Notably, YPF supplementation showed more pronounced benefits in mitigating intestinal oxidative damage compared to hemolymph results, with the 1000P group exhibiting even higher antioxidant enzyme activities than the CT group. This suggests that 1000mg/kg YPF has a significant positive impact on optimizing the LF diet by improving intestinal oxidative damage. These superior repair effects of YPF on intestinal oxidative damage may be attributed to its enhancement of the intestinal barrier, modulation of immune cells, and influence on gut microbiota metabolism (39–41).

Our initial focus is on exploring the function of the intestinal barrier. Ultrastructural analysis revealed that the LF diet significantly impaired intestinal morphology, including damage to the villi and basal membrane. Notably, the peritrophic matrix (PM), a semi-permeable membrane unique to invertebrate intestines, exhibited detachment and degradation under the LF diet (42). Transmission electron microscopy further confirmed this structural damage, showing a lack of tightly attached PM on the microvilli in the LF diet group. The PM, composed mainly of chitin and proteins, plays a crucial role in protecting intestinal epithelial cells from physical damage and facilitating nutrient digestion and absorption (43). Previous research has linked PM disruption with intestinal oxidative stress and functional impairment (44, 45). YPF supplementation in the LF group significantly alleviated PM damage, demonstrating a protective effect on maintaining intestinal PM structure. The synthesis of chitin, a key component of PM, relies on chitin synthase (CHS) (46) and glutamine synthetase (GS), which provides the necessary nitrogen source, glutamine, for chitin synthesis (47). Eritrophin-like protein (EcPT) has also been shown to maintain PM stability and function in prawns (48). Our study found that the LF diet significantly suppressed the expression of these three key PM-related genes, corroborating that the LF diet caused PM damage. Additionally, we assessed the levels of LZM and LPS, which are associated with PM function, finding that the LF group exhibited downregulated LZM and elevated LPS levels, a phenomenon also indicative of PM damage (13, 49). YPF supplementation notably improved this condition, especially at 1000mg/kg YPF, further validating the protective effect of YPF on PM function. The decrease in LZM and increase in LPS suggest that the LF diet may exacerbate intestinal dysfunction (50), while YPF may restore PM integrity through various mechanisms, including modulation of cellular interactions and providing antioxidant and immune protection (51). Consequently, it is warranted to investigate how YPF polysaccharide supplementation can further enhance intestinal immunity under a low-fishmeal diet.

In examining the immune responses of *M. rosenbergii* in this study, we focused on non-specific immune capacity regulation, as it is widely accepted that crustaceans do not exhibit inflammatory responses and lack a specific immune system akin to mammals. In invertebrate non-specific immunity, the NF-κB pathway plays a crucial role, with IMD-Relish and Toll-Doral being the main homologs in this signaling pathway (52). Our results showed that the LF diet primarily affected the IMD-Relish pathway, suppressing its expression levels. Studies indicate that inhibition of the IMD-Relish pathway may exacerbate oxidative stress and impact overall host health and immune function (28). The up-regulation of IκBα in the LF group supports this, as increased IκBα indicates suppressed NF-κB pathway activity and diminished innate immune response (53). Additionally, research has shown that NO produced by iNOS can clear oxygen radicals and inhibit lipid peroxidation (54). The activation of the NF-κB signaling pathway (IMD-Relish) being a key step in increasing iNOS activity and inducing NO release (55). The LF group exhibited suppressed IMD-Relish pathway activity and decreased iNOS and NO levels, which aligns with this understanding, suggesting that inhibiting the NF-κB/NO signaling pathway may be a causative factor in oxidative stress induced by the LF diet. And supplementing with 1000mg/kg YPF activated the IMD-Relish pathway and regulated iNOS and NO levels, thereby improving non-specific immune capacity compromised by the LF diet.

Recent attention has been drawn to the significant role of gut microbiota in enhancing intestinal health and immunity in aquatic animals (56). Our study found that the intestinal microbiota composition in LF group prawns differed markedly from the other groups, with a decline in microbial diversity. A diverse gut microbiota promotes nutrient absorption, helps maintain intestinal barrier function, and establishes the intestinal immune system (57). The results from the LF group indirectly reflect deficiencies in intestinal health. To delve deeper into the impact of gut microbiota, we analyzed the composition of each group, finding that the LF diet increased Proteobacteria levels and decreased Firmicutes levels, suggesting a potential negative health state in the host (58). Further analysis of bacterial genera revealed that the LF group experienced a disruption in gut microbial balance, with pathogenic bacteria such as *Pseudomonas*, *Aeromonas*, and *Klebsiella* proliferating, while the levels of probiotic bacteria like *Lactococcus* and *Lactobacillus* significantly decreased (59). Supplementing with 1000mg/kg YPF reversed these phenomena, indicating that YPF may regulate microbiota balance by fostering the growth of beneficial bacteria and curbing the proliferation of harmful bacteria, thus restoring microbial diversity and stability. These enhancements may contribute to strengthening the intestinal barrier and immune function. Several studies corroborate our findings, showing that YPF polysaccharides possess prebiotic potential and positively impact the composition of the intestinal microbiota, increasing the abundance of beneficial bacteria in the intestine (21, 60). Moreover, these studies highlight a strong correlation between the modulation of intestinal microbiota by YPF polysaccharides and the promotion of intestine health, as well as the prevention of gut-related disorders (61). Further investigation is needed to elucidate the effects of gut microbiota alterations on the intestinal barrier (PM) and immunity, and to explore their interaction mechanisms, which are crucial for intestinal health and pathogen defense.

Analyzing the functional aspects of intestinal microbiota, we found that changes in the LF group’s microbial community altered microbiota function, affecting intestinal barrier function and immunity. This analysis revealed a significant reduction in microbial taxa involved in amino acid metabolism and cell motility, suggesting decreased competition for host nutrients. This reduction likely leads to reduced immune stimulants and affects immune cell activity. Supporting evidence includes studies showing a correlation between microbial competition for nutrients and immune stimulant production, such as Puig et al. (2015), which demonstrated that intestinal microbial competition influences the host’s immune response (62). In contrast, the 1000P group’s microbiota exhibited enhanced oxidative phosphorylation, glycolysis/gluconeogenesis, the citric acid cycle, and purine metabolism, improving energy utilization efficiency. This enhancement may produce beneficial metabolites such as short-chain fatty acids (SCFAs) and bile acids, strengthening PM integrity and intestinal immunity, and positively impacting intestinal health (63, 64).

Subsequent correlation analysis between distinct microbial genera and intestinal health factors revealed close associations between certain intestinal microbes and PM formation and intestinal innate immunity. *Lactobacillus* showed a significant positive correlation with PM formation factors, suggesting that this microbe may contribute to maintaining PM, likely by protecting intestinal epithelial cells through SCFA production and enhancing PM barrier function (65). Conversely, *Chitinibacter*, which negatively correlated with PM factors, is prevalent in chitin-rich environments and degrades chitin (primary component of PM) (66), implying that *Chitinibacter* may negatively impact PM formation by degrading chitin. *Lactobacillus, Chitinibacter*, and *Lactococcus* significantly correlated with indicators of non-specific immunity. *Lactobacillus* and *Lactococcus* are known for supporting intestinal immunity through NF-κB pathway regulation, antimicrobial substance production, and interactions with the host immune system (67, 68). In contrast, *Chitinibacter*’s specific role remains unclear but may indirectly influence intestinal immunity by affecting PM formation. Network analysis identified *Lactobacillus* and *Chitinibacter* as key regulators of intestinal PM and non-specific immunity, suggesting that LF diet and YPF supplementation may modulate intestinal immune status through these microbes. For further studies, multi-omics and *in vivo* approaches are necessary to elucidate whether these microbes influence intestinal health through metabolic products or direct immune regulation (69).

## 5 Conclusions

In summary, the low-fishmeal (LF) diet may impair the peritrophic matrix (PM) and intestinal immunity by increasing *Chitinibacter* and decreasing *Lactobacillus*, leading to compromised intestinal health and oxidative stress of *M. rosenbergii*. YPF supplementation may mitigate the effects of the LF diet on these microbial populations, thereby protecting intestinal health, especially at a concentration of 1000mg/kg YPF.

## Data Availability

The raw dataset for 16SrDNA sequencing have been deposited in the National Institutes of Health’s Short Read Archive database (SRA accession no. PRJNA1048554).
