# Supplementary material for: Supplementation of Yupingfeng polysaccharides in low fishmeal diets enhances intestinal health through influencing the intestinal barrier, immunity, and microflora in Macrobrachium rosenbergii
Source: Front Immunol. 2024 Nov 26;15:1480897. doi: 10.3389/fimmu.2024.1480897 (PMC11628508; doi:10.3389/fimmu.2024.1480897)
Supplement: Supplementary file 1 [file Table1.docx]

**Table S1.** The growth evaluation of *M. rosenbergii*

| YPF levels (mg/kg) | Index | | | | | |
| --- | --- | --- | --- | --- | --- | --- |
|  | SR (%) | IW (g) | FW (g) | WGR (%) | SGR (%/day) | FCR |
| 0 | 87.50±4.24 | 0.19±0.01 | 2.90±0.12^a^ | 1493.72±21.78^a^ | 6.08±0.05^a^ | 1.33±0.04^b^ |
| 50 | 84.17±3.52 | 0.20±0.01 | 3.05±0.09^ab^ | 1516.26±18.62^ab^ | 6.17±0.12^ab^ | 1.15±0.01^a^ |
| 100 | 85.00±3.70 | 0.20±0.00 | 3.24±0.08^b^ | 1538.12±24.57^ab^ | 6.15±0.14^ab^ | 1.25±0.02^b^ |
| 200 | 86.70±4.23 | 0.19±0.01 | 3.21±0.11^b^ | 1528.22±21.62^ab^ | 6.32±0.06^b^ | 1.18±0.06^ab^ |
| 500 | 86.3±4.46 | 0.20±0.01 | 3.46±0.14^c^ | 1610.14±20.10^b^ | 6.48±0.08^c^ | 1.16±0.03^a^ |
| 1000 | 85.8±2.46 | 0.20±0.00 | 3.42±0.06^c^ | 1598.87±15.44^b^ | 6.43±0.15^bc^ | 1.15±0.03^a^ |

Notes: SR, survival rate; IW, Initial weight; FW, final weight; WGR, weight gain rate; SGR, specific growth rate; FCR, Feed conversion ratio.

**Table 2.** Biochemical indicators in hemolymph of *M. rosenbergii*

| YPF levels (mg/kg) | Index | | | | |
| --- | --- | --- | --- | --- | --- |
|  | AST | ALT | TP | ALB | GLB |
| 0 | 40.22±2.37^c^ | 18.34±0.87b | 20.52±1.14^b^ | 8.82±0.34 | 15.58±0.65^a^ |
| 50 | 34.70±2.18^b^ | 17.42±1.21^b^ | 21.04±2.09^b^ | 8.70±0.40 | 14.97±1.06^a^ |
| 100 | 33.52±1.75^b^ | 18.05±1.82^b^ | 20.26±1.09^b^ | 8.07±0.49 | 15.06±0.72^a^ |
| 200 | 32.02±1.61^ab^ | 16.78±1.29^b^ | 19.04±1.34^ab^ | 7.87±0.82 | 16.34±0.55^ab^ |
| 500 | 27.82±3.10^a^ | 14.07±0.72^a^ | 17.48±1.68^a^ | 8.12±0.27 | 17.62±0.37^b^ |
| 1000 | 28.21±1.08^a^ | 13.44±1.02^a^ | 18.24±0.82^a^ | 8.18±0.08 | 17.06±0.12^b^ |

Notes: AST, aspartate aminotransferase; ALT, alanine transaminase; TP, total protein; ALB, albumin; GLB, globulin.
